# Bidirectional Mendelian Randomization Analysis of Genetic Proxies of Plasma Fatty Acids and Pre-Eclampsia Risk

**DOI:** 10.3390/nu16213748

**Published:** 2024-10-31

**Authors:** Jingqi Zhou, Shuo Jiang, Dangyun Liu, Xinyi Li, Ziyi Zhou, Zhiheng Wang, Hui Wang

**Affiliations:** 1 School of Public Health, Shanghai Jiao Tong University School of Medicine, Shanghai 200025, China; jingqizhou@sjtu.edu.cn (J.Z.); hooleejnn@sjtu.edu.cn (S.J.); lxy2021@sjtu.edu.cn (X.L.); 184739@shsmu.edu.cn (Z.Z.); 2 Department of Central Laboratory, The Affiliated Huaian No. 1 People’s Hospital of Nanjing Medical University, Huai’an 223300, China; hayyldy@njmu.edu.cn; 3 Clinical Laboratory, Obstetrics and Gynecology Hospital of Fudan University, Shanghai 200090, China

**Keywords:** fatty acids, pre-eclampsia, mendelian randomization analysis

## Abstract

Background: Previous studies have reported associations between fatty acids and the risk of pre-eclampsia. However, the causality of these associations remains uncertain. This study postulates a causal relationship between specific plasma fatty acids and pre-eclampsia or other maternal hypertensive disorders (PE-HTPs). To test this hypothesis, two-sample bidirectional Mendelian randomization (MR) analyses were employed to determine the causality effects. Methods: Single-nucleotide polymorphisms associated with PE-HTPs and fatty acids were obtained from a genome-wide association study (GWAS) of European ancestry. Bidirectional MR analyses were conducted using methods such as inverse variance weighted, MR-Egger, weighted median, simple mode, and weighted mode. Sensitivity analyses, including tests for heterogeneity, horizontal pleiotropy, and co-localization, were conducted to assess the robustness of MR results. Results: The analyses revealed causal relationships between PE-HTPs and several fatty acids, including monounsaturated fatty acid (MUFA), omega-6 fatty acid (*n*-6 FA), linoleic acid (LA), docosahexaenoic acid (DHA), and the PUFA/MUFA ratio. Genetically predicted higher risk of PE-HTPs was significantly associated with lower plasma *n*-6 FA (OR = 0.96, 95% CI: 0.93–0.99), particularly LA (OR = 0.95, 95% CI: 0.92–0.98). Conversely, increased DHA (OR = 0.86, 95% CI: 0.78–0.96) and a higher PUFA/MUFA ratio (OR = 0.86, 95% CI: 0.76–0.98) were associated with a reduced risk of PE-HTPs. Elevated MUFA levels (OR = 1.12, 95% CI: 1.00–1.25) were related to an increased risk. Conclusions: This study provides robust genetic evidence supporting bidirectional causal relationships between PE-HTPs and specific plasma fatty acids, underscoring the critical role of fatty acid metabolism in maternal hypertensive disorders.

## 1. Introduction

Pre-eclampsia is a common pregnancy complication characterized by hypertension and proteinuria [1], affecting 3% to 5% of all pregnancies [1,2]. Hypertensive disorders of pregnancy, including pre-eclampsia, chronic hypertension, and gestational hypertension, impact approximately 10% of pregnancies [3]. Pre-eclampsia poses significant risks to both maternal and fetal health, notably impacting fetal cognitive function such as verbal reasoning and executive function in early childhood [4]. Research suggests that pre-eclampsia may influence long-term brain and mental health, with studies indicating potential variations in the structure and connectivity of the limbic system components [5,6,7]. Additionally, pre-eclampsia is often associated with defects in placental development and function, with susceptibility potentially driven by underlying cardiometabolic factors that modify responses to pregnancy-induced stress [8,9].

Plasma fatty acids play a crucial role in maternal cell growth and development during pregnancy, as well as in cell signaling [10]. They act as both structural components and functional regulators, determining maternal fatty acid metabolism, which is essential for fetal–placental development [11]. In particular, the preferential transport of long-chain polyunsaturated fatty acids (PUFAs) such as docosahexaenoic acid (DHA) and linoleic acid (LA) in plasma is critical for fetal brain and retina development [12,13]. An elevated omega-6-to-omega-3 PUFA ratio (*n*-6/*n*-3 ratio) contributes to a pro-inflammatory state in the body, promoting non-communicable chronic diseases [14], increasing mortality from cardiovascular disease, and affecting cognitive function in children [15,16], among other health implications. In addition, excess dietary saturated fatty acids (SFAs) and trans fatty acids are associated with cardiovascular disease risk [17], and a higher ratio of polyunsaturated to monounsaturated fatty acids (PUFA/MUFAs ratio) is linked to a lower risk of cardiovascular mortality and better overall health outcomes [18]. Women with pre-eclampsia are reported to be susceptible to lipid metabolism disorders. Maternal hypertension during pregnancy can alter placental fatty acid transport, which may be caused by the quality and quantity of fatty acids [19,20,21]. Although the specific mechanism behind these placental changes in lipid transport and metabolism remains unclear, alterations in fatty acids may contribute to changes in fetal brain development in pregnant women with pre-eclampsia.

Most studies on plasma fatty acids in pregnant women with pre-eclampsia or other maternal hypertensive disorders (PE-HTPs) employ case–control or cross-sectional designs [21,22,23,24,25,26]. While these methodologies offer valuable insights, they inherently limit the ability to infer causal relationships due to their observational nature and potential for confounding variables. To address these limitations, our study used Mendelian randomization (MR) to evaluate the consistency of correlations with the causal hypothesis. MR leverages genetic variation as instrumental variables in observational research, simulating randomized controlled trials through the random assignment of alleles to offspring at meiosis, thereby mitigating the influence of potential confounders on exposures and controls [27]. This approach enables the detection and quantification of causality within observational studies. In this study, genetic proxies for various types of plasma fatty acids were derived from genetic variations, while PE-HTP outcomes were obtained from large biobanks. Our aim was to use bidirectional MR to assess the causal association between plasma fatty acid levels and the risk of pre-eclampsia. This research will provide a scientific foundation for future mechanistic and interventional studies aimed at the prevention and treatment of pregnancy-related comorbidities.

## 2. Materials and Methods

### 2.1. Study Design

**Figure 1:**
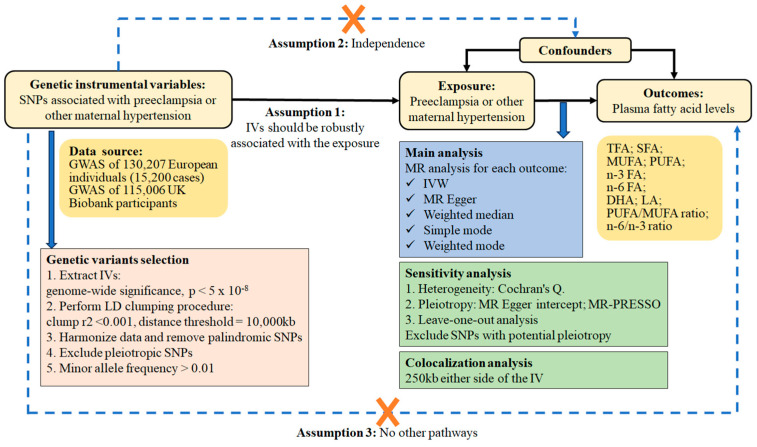
Flowchart of the study design. Abbreviations: DHA, docosahe×aenoic acid; GWAS, genome-wide association study; IV, instrumental variable; IVW, inverse variance weighting; LA, linoleic acid; LD, linkage disequilibrium; MR, Mendelian randomization; MR-PRESSO, MR pleiotropy residual sum and outlier; MUFA, monounsaturated fatty acid; *n*-3 FA, omega-3 fatty acid; *n*-6 FA, omega-6 fatty acid; PUFA, polyunsaturated fatty acid; SFA, saturated fatty acid; SNP, single-nucleotide polymorphism; TFA, total fatty acid.

MR analysis is predicated on three key assumptions. It is imperative to emphasize that MR should exclusively incorporate objective assessments, unless explicitly denoted as subjective. Firstly, the genetic variants serving as instrumental variables (IVs) must be strongly correlated with the exposure trait. Secondly, these IVs must not be subject to any confounding factors. Lastly, genetic IVs do not directly influence the outcome of circulating fatty acids but rather exert their impact solely through indirect exposure pathways. In this study, we employed a two-sample MR approach to examine the impact of PE-HTPs on circulating fatty acids utilizing summary statistics from genome-wide association studies (GWAS) [28,29]. In order to minimize the impact of population stratification, the present study was limited to participants with European ancestry. Ten plasma fatty acid-related outcomes were selected, including total fatty acids (TFAs), SFAs, monounsaturated fatty acids (MUFAs), and PUFAs. Additionally, omega-3 fatty acids (*n*-3 FAs) and omega-6 fatty acids (*n*-6 FAs) were included, as well as the main constituent fatty acids: DHAs and LAs. Two ratios were also added as outcomes: the PUFA/MUFA ratio and the *n*-6/*n*-3 ratio. Furthermore, a reverse MR analysis was performed to determine the causal impact of the aforementioned fatty acids on PE-HTPs. The study design is illustrated in Figure 1.

### 2.2. Data Sources and Study Population

PE-HTP is a combined phenotype including pre-eclampsia or other maternal hypertension. Briefly, the PE-HTP GWAS was derived from a meta-analysis combining data from the Finnish Genetics of Preeclampsia Consortium (FINNPEC, 1990–2011), the Finnish FinnGen project (1964–2019), the Estonian Biobank (EstBB, 1997–2019), and the previously published InterPregGen Consortium GWAS [28]. We used summary statistics downloaded from the NHGRI-EBI GWAS Catalog [30] on 19 February 2024 to examine the association between genetic variants and plasma levels of fatty acids [29]. Fatty acid data were selected from 249 metabolic traits measured by targeted high-throughput nuclear magnetic resonance (NMR) metabolomics by Nightingale Health (biomarker quantification version 2020) in the UK Biobank. Following the original GWAS, each fatty acid trait was normalized to have a mean of 0 and a standard deviation of 1, using inverse rank normalization, to allow for comparisons between derived effect estimates [29]. Further details were well described in previous studies [31]. Ethical approval was obtained for all original studies.

### 2.3. SNP Selection

We selected single-nucleotide polymorphisms (SNPs) associated with PE-HTPs (*p* < 5 × 10^−8^) in the previous GWAS meta-analysis in individuals of European ancestry. Independent variants (r^2^ < 0.01 and kb > 5000) were selected using the “clump_data” function (EUR population) of the “TwoSampleMR” R package to remove genes in linkage disequilibrium (LD) [32]. Next, we eliminated IVs significantly associated with plasma fatty acid phenotypes to ensure compliance with Assumption 3. The F-statistic for each SNP was estimated as the square of the SNP-exposure association divided by the variance of the SNP-exposure association. IVs with F-statistics below 10 are deemed weak, and thus excluded.

### 2.4. MR Analyses

We mainly used random effects with inverse variance weighting (IVW) and multiplication, assuming pleiotropic equilibrium. The presence of heterogeneity due to pleiotropy was indicated by high Cochran’s Q and I^2^ statistics. To ensure consistency in the use of alleles for both exposure and outcome for palindromic SNPs (coded A/T or C/G), we aligned them based on their effect allele frequency and coding directionality. In sensitivity analyses, we also included MR estimates from alternative methods with different assumptions, including the weighted median, MR-Egger, weighted mode and simple mode approaches. In addition, we used MR pleiotropy residual sum and outlier (MR-PRESSO) analysis and leave-one-out (LOO) sensitivity tests as an additional means of identifying potential horizontal pleiotropic outliers and correcting for observed pleiotropy if necessary. Enumerative colocalization analysis was used to evaluate instrumental variable hypotheses for specific genetic regions within this study. If PE-HTP and fatty acid phenotypes were causally influenced by different variants related to each other, this could violate Assumption 3 in the MR analysis by providing a pathway between the genetic variants and outcomes other than via exposure. For example, a genetic predictor of exposure could be in LD with another variant that independently influenced the outcome [33]. All statistical analyses were conducted using R version 4.3.1 and the “MR-PRESSO”, “TwoSampleMR”, and “coloc” packages. Statistical significance was defined as *p* < 0.05.

## 3. Results

### 3.1. Study Population

The PE-HTP GWAS included up to 130,207 individuals of European ancestry from the FINNPEC (1689 cases, 778 controls), FinnGen (9427 cases, 78,601 controls), and EstBB (4048 cases, 35,628 controls) cohorts [25]. According to the previous study, the mean ± SD age at diagnosis of PE-HTP for the FINNPEC, FinnGen, and EstBB cohorts was 30.3 ± 5.5, 29.5 ± 5.8, and 30.2 ± 7.2, respectively. The summary statistics of plasma levels of fatty acids were based on a GWAS of individuals of European ancestry from the UK Biobank (N = 115,006) [29].

### 3.2. Association Between Genetically Estimated PE-HTP and Plasma Fatty Acids

After excluding LD with r^2^ < 0.01, eight SNPs associated with PE-HTPs reached suggestive genome-wide significance (*p* < 5 × 10^−8^). The F-statistics of all instrumental variables ranged from 32 to 54, indicating a relatively low risk of weak instrument bias in MR analyses. Upon removing palindromic SNPs, Supplementary Materials’ S1 presents the summary statistics for the genetic variants related to PE-HTP.

**Figure 2:**
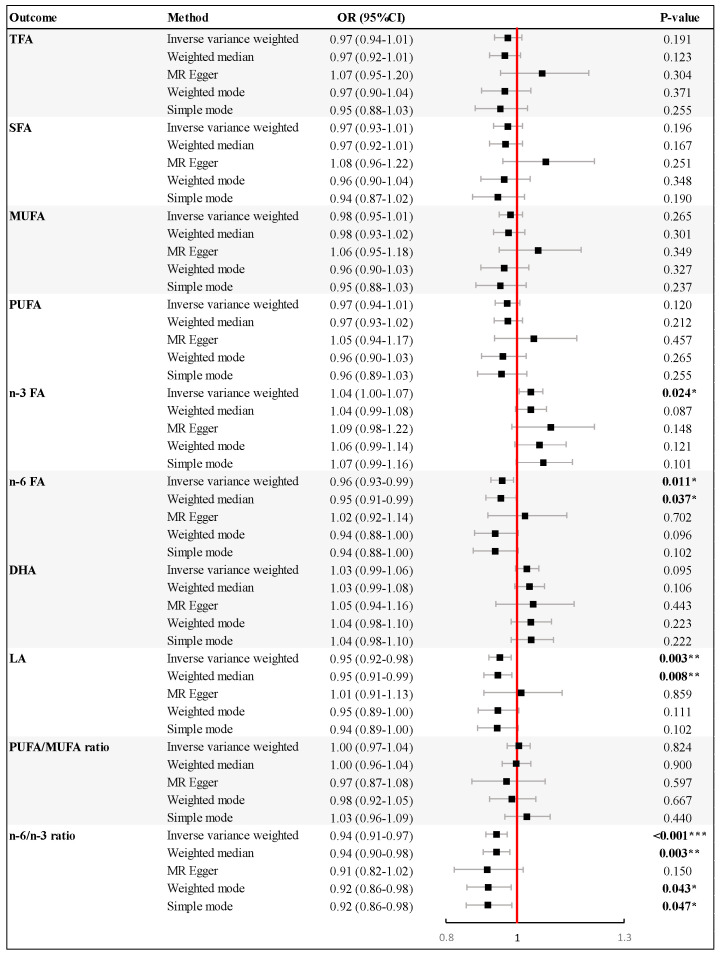
Results of Mendelian randomization (MR) analysis of pre–eclampsia or other maternal hypertensive disorder (PE–HTP) and multiple plasma fatty acids. Forest plots show odd ratios and 95% confidence intervals. The results are shown for the different methods of MR analyses used in this study: inverse variance weighted, weighted median, MR–Egger, weighed mode, and simple mode. *p* values are indicated by stars, * *p* < 0.05, ** *p* < 0.01, *** *p* < 0.001. Abbreviations: CI, confidence interval; DHA, docosahexaenoic acid; LA, linoleic acid; MUFA, monounsaturated fatty acid; *n*-3 FA, omega-3 fatty acid; *n*-6 FA, omega-6 fatty acid; OR, odds ratio; PUFA, polyunsaturated fatty acid; SFA, saturated fatty acid; TFA, total fatty acid.

Figure 2 presents the results of a univariable MR analysis that explored the causal effect of genetically estimated PE-HTPs on various types of plasma fatty acids. In the IVW MR analysis, genetically predicted higher risk of PE-HTPs was related to increased plasma *n*-3 FA levels (odds ratio (OR) = 1.04, 95% confidence interval (CI): 1.00–1.07, *p* = 0.024), and decreased plasma *n*-6 FA levels (OR = 0.96, 95% CI: 0.93–0.99, *p* = 0.011). Specifically, the genetic proxies of PE-HTPs were associated with reduced LA levels (OR = 0.95, 95% CI: 0.92–0.98, *p* = 0.003) for *n*-6 FA. The effect estimate remained significant in the weighted median analyses for *n*-6 FA (OR = 0.95, 95% CI: 0.91–0.99, *p* = 0.037) and LA (OR = 0.95, 95% CI: 0.91–0.99, *p* = 0.008). A higher genetically determined risk of PE-HTP was associated with the *n*-6/*n*-3 ratio in the IVW analysis (OR = 0.94, 95% CI: 0.91–0.97, *p* < 0.001). Besides the IVW method, the weighted median, weighted mode, and simple mode methods obtained *p* values lower than 0.05. However, gene-predicted PE-HTP was not significantly associated with other plasma fatty acids (TFAs, SFAs, MUFAs, and PUFAs) or the PUFA/MUFA ratio, with *p* values above 0.05 calculated by the IVW method and other four methods.

### 3.3. Association Between Genetically Estimated Plasma Fatty Acids and PE-HTPs

**Figure 3:**
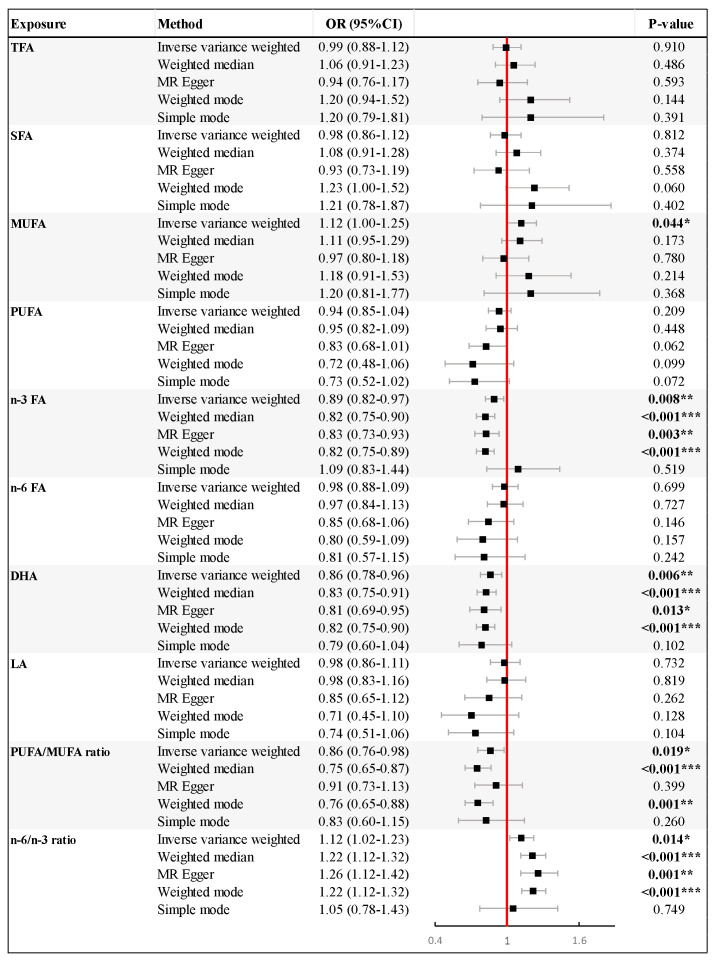
Mendelian randomization (MR) results for effect of genetically predicted fatty acid levels on asthma pre–eclampsia or other maternal hypertensive disorder (PE–HTP). Forest plots show odd ratios and 95% confidence intervals. The results are shown for the different methods of MR analyses used in this study: inverse variance weighted, weighted median, MR–Egger, weighed mode, and simple mode. *p* values are indicated by stars, * *p* < 0.05, ** *p* < 0.01, *** *p* < 0.001. Abbreviations: CI, confidence interval; DHA, docosahexaenoic acid; LA, linoleic acid; MUFA, monounsaturated fatty acid; *n*-3 FA, omega-3 fatty acid; *n*-6 FA, omega-6 fatty acid; OR, odds ratio; PUFA, polyunsaturated fatty acid; SFA, saturated fatty acid; TFA, total fatty acid.

In the reverse MR analysis with the IVW method, higher genetically estimated levels of *n*-3 FA (OR = 0.89, 95% CI: 0.82–0.97, *p* = 0.008) and DHA (OR = 0.86, 95% CI: 0.78–0.96, *p* = 0.006) were found to reduce the risk of PE-HTPs significantly. Additionally, the increased *n*-6/*n*-3 ratio was significantly associated with a higher risk of PE-HTP (OR = 1.12, 95% CI: 1.02–1.23, *p* = 0.014). Similar findings were observed in the weighted median, MR Egger, and weighted analyses, which also showed associations of *n*-3 FA, DHA levels, and *n*-6/*n*-3 ratio with PE-HTPs. Furthermore, the IVW analysis indicated that elevated levels of genetically determined MUFAs increased the risk of PE-HTPs (OR = 1.12, 95% CI: 1.00–1.25, *p* = 0.044), while an increased PUFA/MUFA ratio significantly decreased the risk of PE-HTP (OR = 0.86, 95% CI: 0.76–0.98, *p* = 0.019). However, no association was found between TFAs, SFAs, PUFAs, *n*-6 FAs, and LAs with PE-HTPs (Figure 3).

### 3.4. Evaluation of the Assumptions of MR

For Assumption 1, SNPs associated with PE-HTPs and plasma fatty acids were selected from large-sample GWAS, with a genome-wide significance threshold of *p* < 5 × 10^−8^ strictly. F statistics for each SNP were all greater than 30, which ensures that the selected IVs have strong associations with exposure and avoid instrumental bias. The total proportions of variance (R^2^) in the PE-HTPs explained by their corresponding SNPs was about 0.2%, and 5% to 10% in the fatty acids. In addition, for Assumption 2, we used LDtrait to search for whether the IVs were previously associated with a trait or disease that could be a confounder [34]. A significant association was found between the SNP rs1421085 and the phenotypes of weight, waist circumference, and body mass index (BMI), which may introduce confounding effects. Consequently, rs1421085 was excluded from further analysis, and identical findings were observed. Finally, for Assumption 3, we assessed heterogeneity and pleiotropy, respectively. The results are presented in Table 1. The *p* values for each fatty acid were greater than 0.05 using both the MR-Egger and IVW methods, indicating that the analysis of the association between different fatty acids in plasma by PE-HTP was not affected by heterogeneity. Similarly, the *p* values for each fatty acid in the pleiotropy assessment were also above 0.05, suggesting that the analyses of the individual fatty acid outcomes are not affected by pleiotropy, thus not violating Assumption 3.

**Figure 4:**
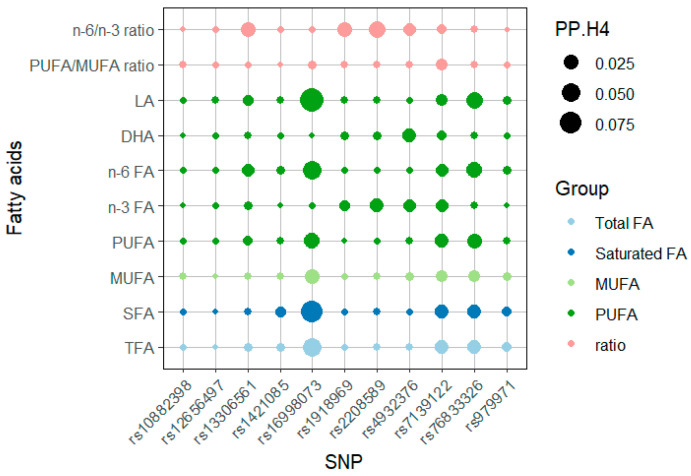
Dot heatmap of posterior probability of H4 (PP.H4) in the colocalization analyses. The eleven independent SNPs associated with PE-HTP were subjected to colocalization analysis with fatty acid traits in plasma. We depict the PP.H4 (evidence of colocalization) in dot size and the group of fatty acids in dot color. Numerical results are shown in Supplementary Materials S2. Abbreviations: DHA, docosahexaenoic acid; LA, linoleic acid; MUFA, monounsaturated fatty acid; *n*-3 FA, omega-3 fatty acid; *n*-6 FA, omega-6 fatty acid; PP.H4, posterior probability of H4; PUFA, polyunsaturated fatty acid; SFA, saturated fatty acid; SNP, single-nucleotide polymorphism; TFA, total fatty acid.

In further GWAS-GWAS colocalization analyses for each fatty acid outcome, we examined the genetic region within 100 kilobase pairs on either side of each IV and found that the posterior probability of H1 (PP.H1) explained more than 85% of all the outcomes, whereas the posterior probability of H4 (PP.H4), representing shared variation, was less than 10% for all (Figure 4, Supplementary Data S2). This indicates that the exposure and the outcome were not affected by the same variant, demonstrating that the IVs used in this study were not pleiotropic and were only strongly associated with exposure.

## 4. Discussion

### 4.1. Principal Findings

Combining bidirectional MR analyses revealed no clear causal relationship between PE-HTP and overall plasma fatty acids. However, for specific types of fatty acids, we found that PE-HTP may reduce plasma *n*-6 FA and, particularly, LA. Additionally, MUFAs may be a risk factor for PE-HTP, whereas DHAs and the PUFA/MUFA ratio may have protective roles.

### 4.2. Comparison with Other Studies

Previous studies have suggested that PE-HTPs may cause disturbances in lipid metabolism in pregnant women, affecting plasma fatty acid composition. The findings of previous observational studies have indicated a transient reduction in maternal plasma *n*-3 FA and DHA at 16–20 weeks of gestation in pre-eclampsia, without subsequent alterations [23,25,35]. This study also verified decreased DHA and *n*-3 FA concentrations in umbilical cord blood of affected neonates [23], a phenomenon echoed by additional research [36]. Several intervention trials have been conducted to determine whether supplementation of *n*-3 FAs from fish oil affects maternal outcomes associated with pre-eclampsia, but the results were largely invalid [37]. However, because the ability of fatty acids such as DHA to be transferred across the placenta to the fetus is impaired in patients with pre-eclampsia, we cannot simply assume that the results of existing studies are contrary to our study [38,39,40]. Future studies are needed to determine whether increased prenatal intake of *n*-3 FA by pregnant women improves cord blood DHA levels and developmental outcomes in infants born to pre-eclamptic mothers. Interestingly, previous studies found increased DHA levels in the breast milk of pre-eclampsia women and no significant difference in LA, which may have a similar mechanism to the results of the present study [24,41,42].

LA, an *n*-6 FA, is a precursor for the synthesis of other important fatty acids in the body and is essential for maintaining cell membrane integrity and normal physiological function. Evidence suggests that plasma levels of LA may be significantly affected in patients with pregnancy-induced hypertension (PIH). Studies have shown that pregnant women with pre-eclampsia have decreased plasma LA [43,44] but increased serum LA compared with normal pregnancies [45]. This change may be related to factors such as inflammatory responses, oxidative stress, or abnormalities in vascular endothelial function caused by PIH. Inflammatory responses can often lead to alterations in fatty acid metabolic pathways that may affect the synthesis and release of LA. These studies support our findings on LA changes.

Similar to the reverse MR analyses, a study has used MR analysis to investigate the impact of maternal fatty acid levels on PIH [46]. This study found that increasing *n*-3 FAs and DHAs may reduce the risk of PIH, whereas increasing the *n*-6/*n*-3 ratio may increase the risk of PIH. The effects of other fatty acids, including TFAs, SFAs, and *n*-6 FAs, on PIH were not confirmed by MR analyses. These results are consistent with those of our reverse MR analyses.

### 4.3. Possible Mechanisms

The precise mechanisms through which pre-eclampsia and gestational hypertension affect maternal plasma fatty acid levels remain unclear. Most mechanistic research focuses on *n*-3 fatty acids and DHA, with less exploration of other mechanisms. Alterations in *n*-3 FA transport and metabolism are evident in pre-eclampsia, characterized by reduced *n*-3 FA levels in placental tissue and umbilical cord blood compared to normal pregnancies. In cases of severe eclampsia and gestational hypertension, reduced mRNA expression of MFSD2A (2A containing the major facilitator superfamily structural domain) in the placenta may lead to increased maternal DHA levels and reduced fetal DHA exposure, possibly due to compromised placental transfer mechanisms [12,47]. In addition, differences in seafood intake between pre-eclamptic and normal pregnant women suggest that changes in plasma DHA levels are not entirely attributable to dietary differences [48].

*n*-6 FA is a significant group of unsaturated fatty acids, with LA being the most common representative. These fatty acids play a crucial role in maintaining cell membrane integrity, modulating inflammatory responses, and supporting nervous system function. Numerous studies have investigated the association and potential mechanisms between *n*-6 FA and LA and hypertensive disorders [49,50]. However, the mechanisms through which they are associated with blood-pressure-related disorders during pregnancy remain unknown. Some studies have suggested that consuming adequate amounts of LA may help to lower blood pressure [50]. Conversely, excessive linoleic acid intake may be associated with an increased risk of hypertension. Excessive intake of *n*-6 FA may lead to an inflammatory response, affecting blood vessel function and causing high blood pressure [49]. Therefore, it is important to control the intake of *n*-6 FA and LA while ensuring adequate intake to maintain cardiovascular health.

### 4.4. Strengths and Limitations

The strengths of this study include the use of two-sample MR analysis, which mitigates confounding bias and explores causality. The GWAS meta-data on hypertension in pregnancy was applied, combining different cohorts to obtain a larger sample size and facilitate the utilization of large-scale genetic data on hypertension in pregnancy. Additionally, this study found that PE-HTP had different effects on two PUFAs, *n*-3 FA and *n*-6 FA.

However, this study has some limitations. The dataset primarily used in our study predominantly comprises individuals of European ancestry, a strategic choice that was made to minimize the confounding effects of ethnicity on our findings. While this approach has strengthened the internal validity of our results, it may somewhat limit the generalizability of our conclusions to populations with different ancestral backgrounds. Additionally, the MR methodology relied on publicly available GWAS summary data, which encompasses a more limited range of fatty acid types and lacks the provision of average values for various density lipoproteins for certain groups. These markers reflect the degree of lipid metabolism, thereby constraining our capacity to conduct a comprehensive investigation into the impact of PE-HTP on a broader spectrum of fatty acid types. Furthermore, it should be noted that the collection and analysis of these data were not specifically intended to investigate the correlation between hypertension and fatty acids during pregnancy, and therefore cannot provide time-series information on the impact of PE-HTP on changes in fatty acids. Finally, due to the nature of the GWAS data, detailed information on lifestyle and environmental factors of individuals was not available to adequately account for potential confounders, such as diet, lifestyle habits, and environmental exposures, which may impact this study’s results. These limitations serve as a reminder to exercise caution when interpreting this study’s results.

## 5. Conclusions and Public Health Implications

In conclusion, our study found that genetic proxies of PE-HTP significantly affect maternal plasma fatty acid composition. In light of clinical trials that have challenged the relationship between PE-HTPs and fatty acids, our findings may support the role of specific fatty acids in the prevention of hypertensive disorders during pregnancy and the potential for dietary intervention in patients with PE-HTPs. Notably, this study showed an increase in *n*-3 FA and a decrease in *n*-6 FA. However, the decrease in *n*-6 FA, specifically LA, suggests that more attention should be given to the modification and roles of *n*-6 FA in patient interventions.

In the future, it will be necessary to gain a better understanding of these relationships through longitudinal studies of large-scale maternal and fetal cohorts. Direct analysis of breast milk components or biochemical markers in the offspring could provide valuable insights into the impact of pregnancy complications on maternal and infant health. Furthermore, it would be beneficial to conduct studies to explore genetic variation in fatty acid metabolism and its impact on pregnancy complications among different populations. Future studies should seek to establish causality and reveal underlying mechanisms through which hypertensive disorders interact with maternal health and fetal development. This will facilitate the development of personalized nutritional strategies to optimize pregnancy outcomes and maternal and child health.

## Figures and Tables

**Table 1:** Results of heterogeneity and pleiotropy testing.

Outcomes	Heterogeneity Test						Pleiotropy Test		
	MR-Egger			IVW			Egger Intercept ^2^	SE	*p*-Value
	Q ^1^	*p*-Value		Q ^1^	*p*-Value				
TFA	6.804	0.339		9.866	0.196		−0.010	0.006	0.151
SFA	7.149	0.307		11.028	0.137		−0.011	0.006	0.121
MUFA	5.042	0.538		7.061	0.423		−0.008	0.006	0.205
PUFA	6.286	0.392		8.216	0.314		−0.008	0.006	0.224
*n*-3 FA	5.041	0.539		6.093	0.529		−0.006	0.006	0.345
*n*-6 FA	5.968	0.427		7.544	0.375		−0.007	0.006	0.256
DHA	2.388	0.881		2.498	0.927		−0.002	0.005	0.751
LA	4.023	0.674		5.323	0.621		−0.006	0.006	0.298
PUFA/MUFA ratio	2.912	0.820		3.342	0.852		0.004	0.006	0.536
*n*-6/*n*-3 ratio	3.594	0.731		3.918	0.789		0.003	0.006	0.590

^1^ In two-sample Mendelian randomization (MR) settings, Cochran’s Q statistic represents the heterogeneity statistic for the MR-Egger and IVW models. When the Q statistic is considerably larger than its degrees of freedom (the number of instrumental variables minus one), it provides evidence for heterogeneity and invalid instrumental variables IVs. ^2^ To address the possibility of horizontal pleiotropy, we utilized the MR-Egger approach, a standard technique in this context. This method is designed to detect horizontal pleiotropy by examining the significance of its intercept. Abbreviations: DHA, docosahexaenoic acid; IVW, inverse variance weighting; LA, linoleic acid; MR, Mendelian randomization; MUFA, monounsaturated fatty acid; *n*-3 FA, omega-3 fatty acid; *n*-6 FA, omega-6 fatty acid; PUFA, polyunsaturated fatty acid; SE, standard error; SFA, saturated fatty acid; TFA, total fatty acid.

## Data Availability

NHGRI-EBI GWAS Catalog (https://www.ebi.ac.uk/gwas/ (accessed on 20 November 2023)); UK Biobank (https://www.nealelab.is/uk-biobank (accessed on 20 November 2023)).

## Supplementary Materials

The following supporting information can be downloaded at: https://www.mdpi.com/article/10.3390/nu16213748/s1, Supplementary Data S1. Summary statistics of genetic variants. Supplementary Data S2. Numerical results of posterior probability of H4 (PP.H4) in the colocalization analyses.
